# Supplementary material for: Sea-ice derived meltwater stratification slows the biological carbon pump: results from continuous observations
Source: Nat Commun. 2021 Dec 15;12:7309. doi: 10.1038/s41467-021-26943-z (PMC8674288; doi:10.1038/s41467-021-26943-z)
Supplement: Supplementary file 1 — Supplementary Information [file 41467_2021_26943_MOESM1_ESM.pdf]

Supplementary Materials for

Sea-ice derived meltwater stratification slows the biogeochemical carbon pump: results from continuous observations

Wilken-Jon von Appen,[1*] Anya M. Waite,[1,2] Melanie Bergmann,[1] Christina Bienhold,[1,3] Olaf Boebel,[1] Astrid Bracher,[1,4] Boris Cisewski,[5] Jonas Hagemann,[1] Mario Hoppema,[1] Morten H. Iversen,[1,6] Christian Konrad,[1,6] Thomas Krumpen,[1] Normen Lochthofen,[1] Katja Metfies,[1] Barbara Niehoff,[1] Eva-Maria Nöthig,[1] Autun Purser,[1] Ian Salter,[1,7] Matthias Schaber,[5] Daniel Scholz,[1] Thomas Soltwedel,[1] Sinhue Torres-Valdes,[1] Claudia Wekerle,[1] Frank Wenzhöfer,[1,3] Matthias Wietz,[1,3] Antje Boetius,[1,3,6]

[*] Corresponding author: Wilken-Jon.von.Appen@awi.de

This PDF file includes:

Tables S1 to S3
Figures S1 to S6

Other Supplementary Materials for this manuscript include the following:

Supplementary Data 1 (separate file Supplementary_Data_1.xlsx): Excel table with data according to Table S1 that is not available online. The file also contains Tables S1, S2, and S3 as separate sheets.

Table S1: **Overview of the used data.**

Included are the instrument type with which the data was collected, the subchapters in which the data are described, the data repositories, the raw temporal resolution of the data, and the figure number where the data are shown.

Parameter	Figure	Derived from	Temporal	Data description	Data at/in
bathymetry	1	IBCAO 30sec V3	n/a	1	https://www.ngdc.noaa.gov/mgg/bathymetry/arctic/grids/version3_0/
sea ice concentration	1, 4A/S1A	AMSR2	1d	1	https://seaice.uni-bremen.de/data/amsr2/asi_daygrid_swath/ https://doi.pangaea.de/10.1594/PANGAEA.898399
surface heat flux	4B/S1B	ERA-Interim	12h	1	https://apps.ecmwf.int/datasets/data/interim-full-daily/levtype=sfc/
surface PAR reduced to 30 m depth using a constant extinction coefficient	5A/S2A	ERA-Interim surface short wave radiation	24h	6	
surface chlorophyll concentration	5B/S2B/S4	Sentinel3A OLCI	8d	1	https://earth.esa.int/web/sentinel/sentinel-data-access
sea ice velocity	2	EUMETSAT OSI SAF	4mon	1	https://data.meereisportal.de/
sea ice export	2	EUMETSAT OSI SAF	1y	1	Data S1
area coverage of stratification regimes	8	FESOM	1d	2	Data S1
modeled mixed layer depth	S6	PWP	12h	2	n/a
shipboard CTD profiles	3	SBE 911+ pressure, temperature, conductivity	-	3	PS107 CTD: https://doi.org/10.1594/PANGAEA.894189 PS107 UCTD: https://doi.org/10.1594/PANGAEA.886146 PS114 CTD: https://doi.org/10.1594/PANGAEA.898694 JR17005 CTD: https://doi.org/10/c4g3
measurement depth	4C/S1C	SBE37 pressure	1h	5	https://doi.pangaea.de/10.1594/PANGAEA.904565
mixed layer depth	4C/S1C	SBE 37 pressure, temperature, conductivity	1h		
potential density	4D/S1D	SBE 37 pressure, temperature, conductivity	1h		
stratification	4E/S1E	SBE 37 pressure, temperature, conductivity	1h		
apparent oxygen utilization	5C/S2C/7	SBE 37 pressure, temperature, conductivity, oxygen	1h		
chlorophyll fluorescence	5B/S2B/7/S4	WetLabs Triplet Eco chlorophyll fluorometer	1h	7	
optical backscattering	5B/S2B	WetLabs Triplet scattering meter	1s	7	
photosynthetically available radiation	5A/S2A	WetLabs Eco PAR	1h	6	
nitrate	5E/S2E/7	Satlantic SUNA Deep	3h	8	
pCO2	5D/S2D/7	Sunburst SAMI-CO2	1h	9	
pH	5D/S2D	Sunburst SAMI-pH	3h		
acoustic mean volume backscattering	6C/S3C/S5	RDI 75kHz LR-ADCP	1h	11	Data S1; https://doi.pangaea.de/10.1594/PANGAEA.904565
multi-frequency acoustic mean volume	S5	ASL AZFP	1h	11	Data S1
sun light duration	5A/S2A	sunrise equation	n/a	6	https://de.mathworks.com/matlabcentral/fileexchange/55509-sunrise-sunset
available PAR in MLD	5A/S2A	PAR and MLD	1d	6	n/a
inorganic nutrients (NO3, Si, PO4, NO2)	5D/S2D	McLane RAS water samples	7d to 31d	8	Data S1 and https://doi.pangaea.de/10.1594/PANGAEA.936749
eukaryotic DNA read frequency	6A/S3A	McLane RAS water samples	7d to 31d	10	2016-2017: https://www.ebi.ac.uk/ena/browser/view/PRJEB43890 2017-2018: https://www.ebi.ac.uk/ena/browser/view/PRJEB43889
bacterial DNA read frequency	6B/S3B	McLane RAS water samples	7d to 31d		2016-2017: https://www.ebi.ac.uk/ena/browser/view/PRJEB43504 2017-2018: https://www.ebi.ac.uk/ena/browser/view/PRJEB43885
POC flux	6D	KUM sediment trap on lander	28d to 31d	12	Data S1
POC flux	6D	oxygen microprobe on crawler	7d to 8d	12	Data S1
sediment volume flux	6D/S3D	KUM sediment traps in	7d to 60d	12	Data S1; photos available in cruise reports of PS107
detritus sea floor areal coverage	6E	camera on lander	12h	13	Data S1
number of megafauna present	6E	camera on lander	12h		

Table S2: **Deployment and recovery details of the moorings.**
The deployment and recovery stations indicate cruise number, station number, and cast number at the respective station.

Mooring name	Longitude	Latitude	Deployment date	Recovery date	Depth [m]	Deployment station	Recovery station
HG-IV-S-1	4°15.71'E	79°01.38'N	2016-07-11	2017-07-27	2542	PS99/070-1	PS107_3-1
HG-IV-S-2	4°15.71'E	79°01.36'N	2017-08-11	2018-07-16	2599	PS107_38-2	PS114_1-1
HG-IV-FEVI-34	4°19.97'E	79°00.00'N	2016-07-11	2017-07-27	2612	PS99/072-1	PS107_3-2
HG-IV-FEVI-36	4°20.02'E	79°00.00'N	2017-08-11	2018-07-16	2609	PS107_38-1	PS114_2-1
Lander-2016	4°06.72'E	79°04.68'N	2016-07-12	2017-07-27	2448	PS99/077-1	PS107_4-1
Lander-2017	4°06.74'E	79°04.68'N	2017-08-11	2018-09-18	2493	PS107_38-7	MSM77_4-9
Lander-2018	4°06.77'E	79°04.81'N	2018-09-27	2019-09-07	2438	MSM77_35-1	PS121_47-1
F4-S-1	6°57.89'E	79°00.71'N	2016-07-10	2017-08-13	1223	PS99/067-1	PS107_44-1
F4-S-2	6°57.86'E	79°00.70'N	2017-08-15	2018-07-18	1260	PS107_49-1	PS114_10-1
F4-17	7°00.03'E	79°00.01'N	2016-07-23	2018-07-18	1218	PS100/018-1	PS114_11-1
F5-17	5°40.12'E	79°00.02'N	2016-07-23	2018-09-06	2100	PS100/019-1	MSM76_198-1

Table S3: **Types, serial numbers, and depths of the instruments on the moorings.**

Mooring	Type	SN	Depth [m]
HG-IV-S-1	RAS	1412809	24
HG-IV-S-1	PAR	491	24
HG-IV-S-1	Triplet	1426	24
HG-IV-S-1	CO2	156	26
HG-IV-S-1	PH	175	26
HG-IV-S-1	SBE37	14011	26
HG-IV-S-1	SBE37	14012	52
HG-IV-S-2	RAS	1433302	29
HG-IV-S-2	PAR	522	29
HG-IV-S-2	Triplet	1551	29
HG-IV-S-2	SUNA	684	29
HG-IV-S-2	CO2	145	31
HG-IV-S-2	PH	165	31
HG-IV-S-2	SBE37	10507	31
HG-IV-S-2	SBE37	13012	57
HG-IV-FEVI-34	ST	2016434	196
HG-IV-FEVI-34	SBE37	9472	253
HG-IV-FEVI-34	LRADCP	3813	409
HG-IV-FEVI-34	ST	2016433	1223
HG-IV-FEVI-36	ST	2012412	201
HG-IV-FEVI-36	SBE37	15436	258
HG-IV-FEVI-36	LRADCP	5373	414
HG-IV-FEVI-36	ST	201524	1228
Lander-2016	ST	20160000	2446
Lander-2017	ST	2017000	2492
Lander-2018	ST	2018000	2436
F4-S-1	RAS	1412805	26
F4-S-1	Triplet	1425	26
F4-S-1	CO2	155	28
F4-S-1	PH	193	28
F4-S-1	SBE37	14010	28
F4-S-1	SBE37	7691	51
F4-S-1	ST	860016	210
F4-S-2	RAS	1412808	23
F4-S-2	PAR	523	23
F4-S-2	Triplet	1552	23
F4-S-2	SUNA	685	23
F4-S-2	PPS	1433301	25
F4-S-2	CO2	169	25
F4-S-2	PH	192	25
F4-S-2	SBE37	13005	25
F4-S-2	SBE37	13006	61
F4-S-2	ST	2012411	212
F4-17	SBE37	13968	250
F4-17	LRADCP	23978	385
F5-17	AZFP	55112	122
F5-17	LRADCP	24014	380

Abbreviation	Device long name
SBE37	Seabird SBE37 Microcat with ODO
LRADCP	RDI 75kHz Long Ranger ADCP
RAS	McLane Remote Access Sampler
PAR	WetLabs EcoPAR
Triplet	WetLabs EcoTriplet
SUNA	Seabird Deep SUNA nitrate
CO2	SAMI CO2
PH	SAMI pH
ST	KUM Sediment trap
AZFP	ASL Acoustic Zooplankton and Fish Profiler

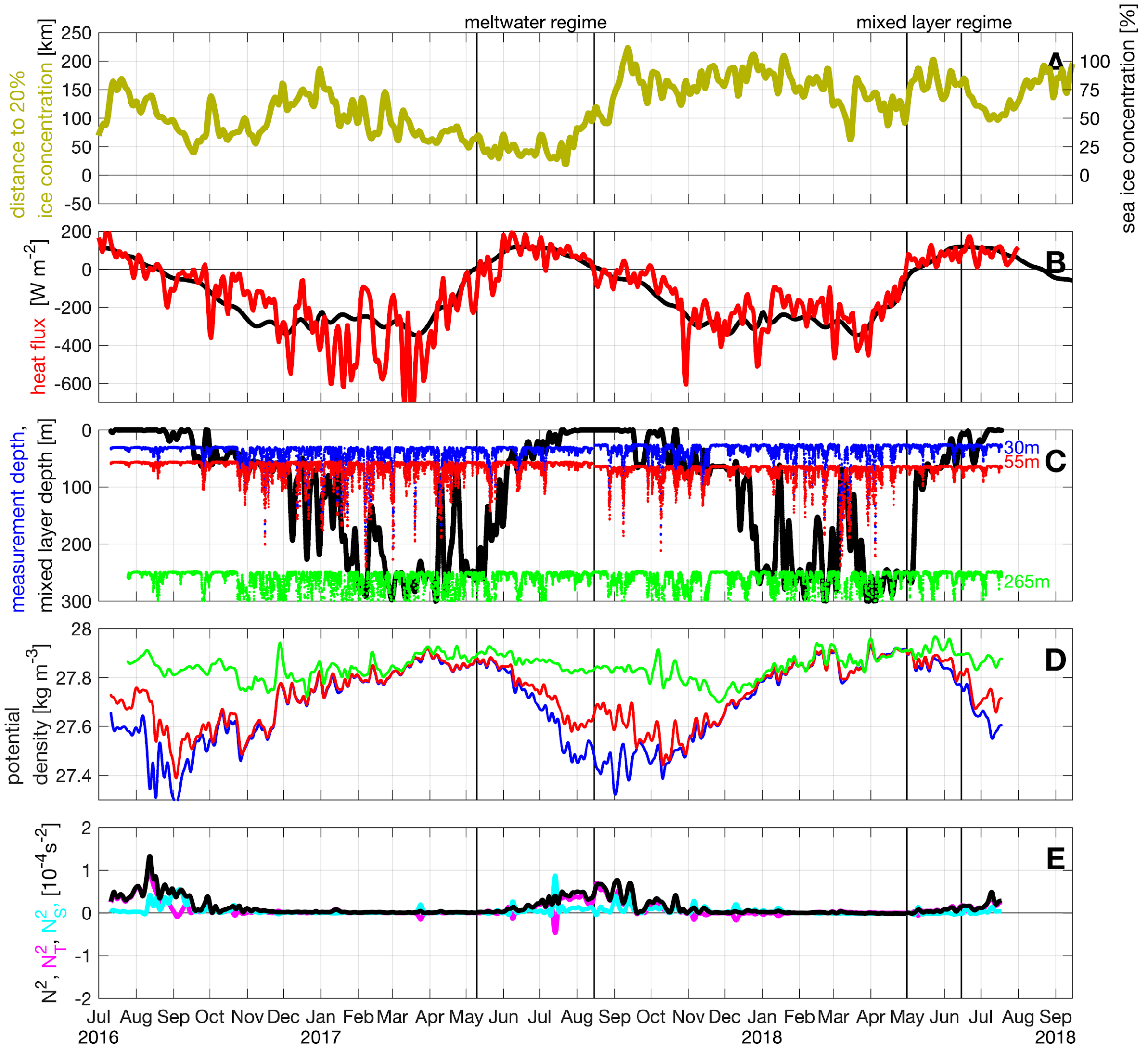

Figure S1: **Ice, heat flux, and upper ocean stratification at F4.**
Same as Fig. 4, but for F4.

a Distance of mooring to 20% sea ice concentration [km] (dark yellow), negative when sea ice concentration at mooring exceeded 20%; sea ice concentration [%] (plotted black when >0%) at AMSR-2 satellite grid point (~5 km diameter) closest to mooring location; note the different y-scale for the sea ice concentration.

b Net surface heat flux [W m^{-2}] (red) at ERI-I reanalysis grid point (~30 km diameter) closest to mooring location and its smoothed climatology (black); positive values warm the ocean.

c Measurement depths of sensors [m] at approximately 30 m (blue), 55 m (red), and 265 m (green); minimum estimate of the mixed layer depth [m] (black), actual mixed layer depth was likely somewhat deeper.

d Potential density [kg/m^3] at the three measurement depths as colored in Fig. S1c.

e Stratification (buoyancy frequency N^2 [s^{-2}], black) estimated from difference of 30 m and 55 m observations and temperature (N^2_T, magenta) and salinity (N^2_S, cyan) contributions to stratification. The time periods of the bloom at HG-IV are marked by vertical black lines for comparison.

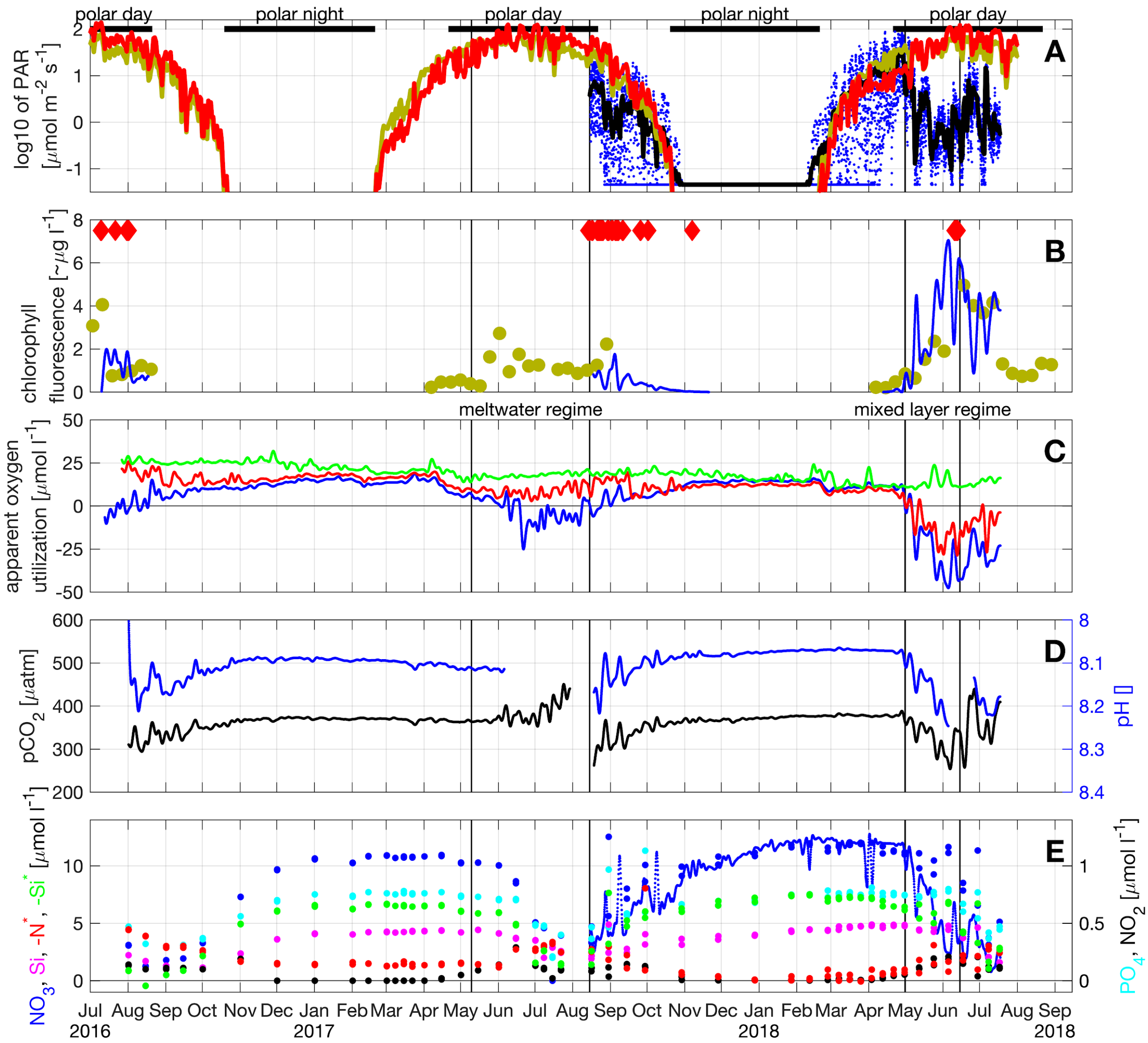

Figure S2: **Upper ocean biogeochemistry at F4** from upper instrument depth (~30 m). Same as Fig. 5, but for F4.

a Logarithm of photosynthetically available radiation [μmol m^{-2} s^{-1}] from hourly sensor measurements at depth (blue) and their 5-day average (black); from reanalysis at the surface reduced to 30 m depth using a constant extinction coefficient in order to make it comparable to the sensor measurements (dark yellow); and from the reanalysis value distributed over the mixed layer depth (red).

b Chlorophyll *a* concentration [~μg l^{-1}] from chlorophyll fluorescence sensor measurements (blue); and from average of Sentinel 3A OLCI satellite grid points within 30-km radius of mooring location (dark yellow); times when individual 1-second measurements of scattering exceeded 0.002 m^{-1} sr^{-1} (red diamonds) indicative of aggregation.

c Apparent oxygen utilization [μmol l^{-1}] at the three measurement depths as colored in Fig. S1c.

d pCO_2 [μatm] (black) and pH [] (blue); note the reversed y-axis for pH.

e Inorganic nutrients [μmol l^{-1}] from water samples in 2016-2017 and from nitrate sensor in 2017-2018; nitrate (blue), silicate (magenta), phosphate (cyan), nitrite (black); negative of (N*=nitrate-phosphate*16) (red); negative of (Si*=silicate-nitrate) (green); note the different y-scale for phosphate and nitrite.

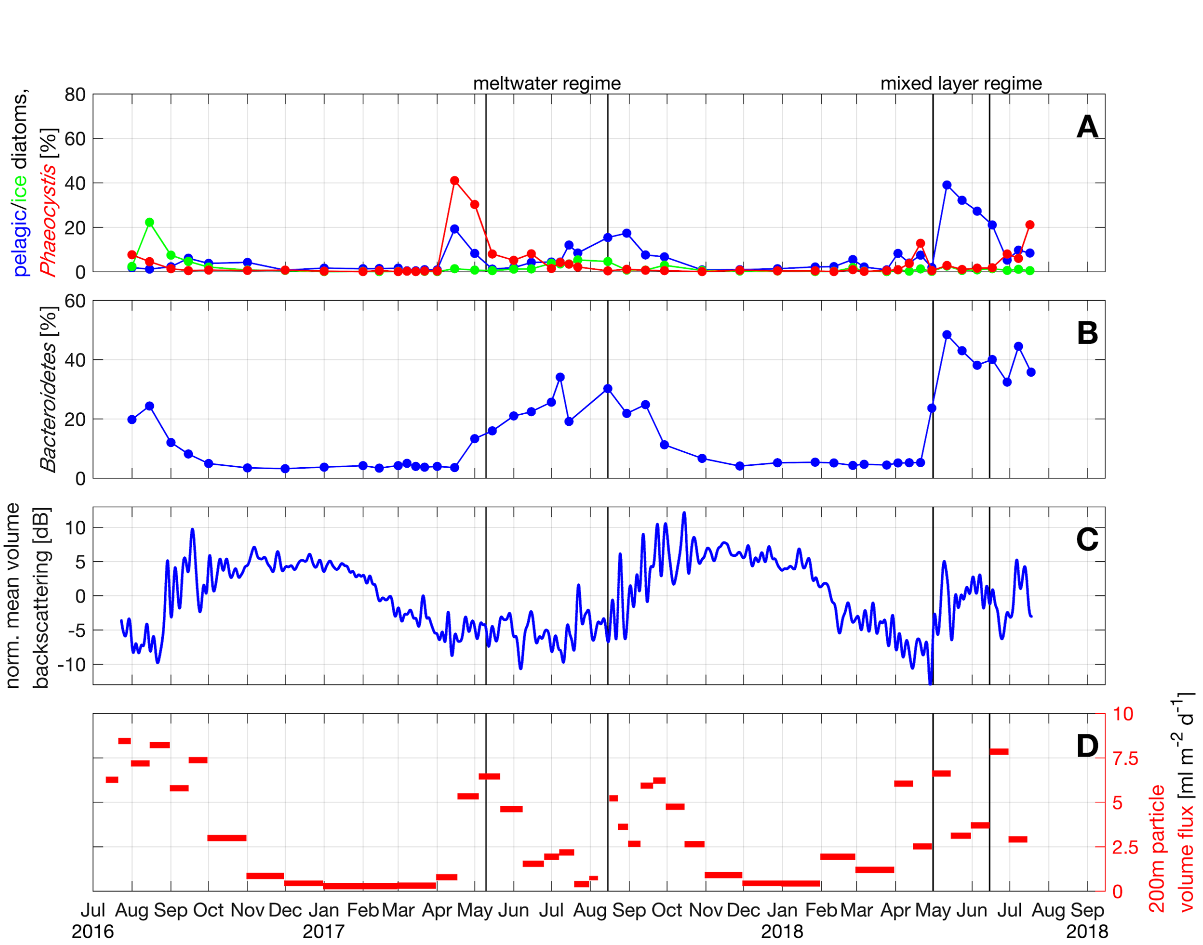

Figure S3: **Upper ocean biology and export at F4.**

Same as Fig. 6a-d, but for F4. Note that no benthic records exist at F4 and export only exists from the sediment trap moored at 200 m depth.

a Relative sequence abundances [% eukaryotes] of pelagic (*Thalassiosira* and *Chaetoceros*; blue) and ice-associated (*Fragilariopsis*; green) diatoms and *Phaeocystis* (red) at ~30 m.

b Relative sequence abundance [% bacteria] of *Bacteroidetes* at ~30 m.

c Normalized mean volume acoustic backscattering [dB] from 75kHz ADCP (blue) averaged over 50-100 m depth range as a proxy for zooplankton biomass; see Fig. S5 for a comparison to shallower reaching multi-frequency AZFPs.

d Particle volume flux [ml m^{-2} d^{-1}] from traps in 200 m depth (red).

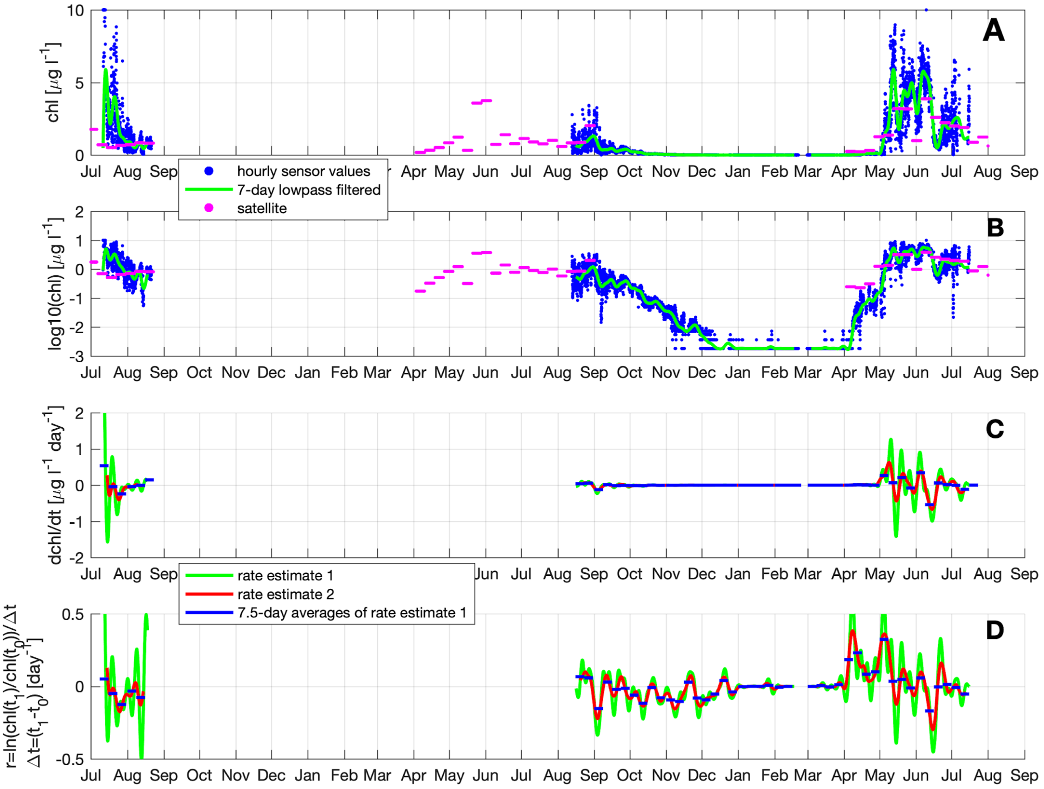

Figure S4: **Growth rates based on chlorophyll *a*** at HG-IV.

A/B Measured chlorophyll *a* concentration at HG-IV [μg l^{-1}] (**A**: linear, **B**: logarithmic base 10) from in-situ sensor (blue, green) and satellite (Sentinel 3A OLCI) (magenta).

C Rate of change of chlorophyll *a* concentration [μg l^{-1} day^{-1}].

D e-folding growth rate based on chlorophyll *a* concentration [day^{-1}] calculated as $r = \ln(chl(t_1)/chl(t_0)) / (t_1-t_0)$. In **C/D** the rates are estimated as the hourly derivative of the 7-day lowpass filtered sensor values (estimate 1) and as 7-day centered differences of the 7-day lowpass filtered sensor values (estimate 2). Note that the chlorophyll sensor did not record data between the end of August 2017 and mid-August 2018.

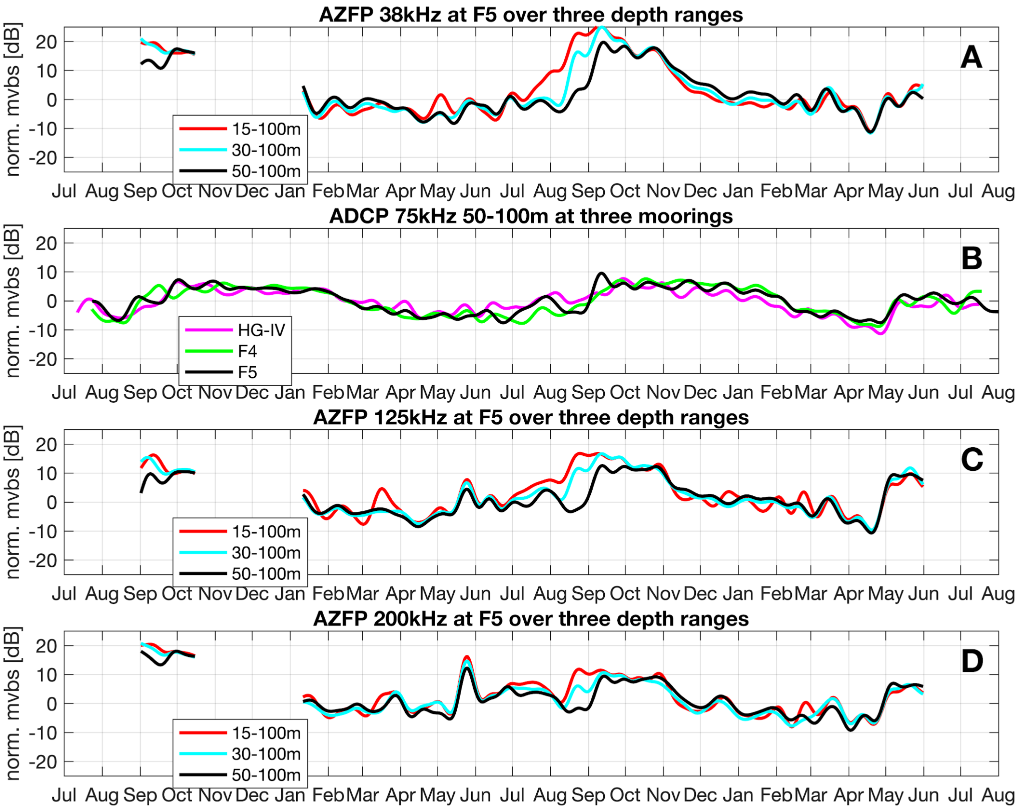

Figure S5: **Acoustic backscatter as proxy for zooplankton and fish concentrations.** Normalized mean volume backscattering from the available acoustic instruments as a proxy for zooplankton biomass.

A/C/D AZFP (Acoustic Zooplankton and Fish Profiler) at F5 (located halfway between moorings HG-IV and F4, Tab. S2) at three frequencies (38 kHz, 125 kHz, 200 kHz) averaged over three different depth ranges (15-100 m, 30-100 m, 50-100 m)

B ADCPs (Acoustic Doppler Current Profiler) at 75 kHz frequency at the three moorings averaged over the 50-100 m depth range.

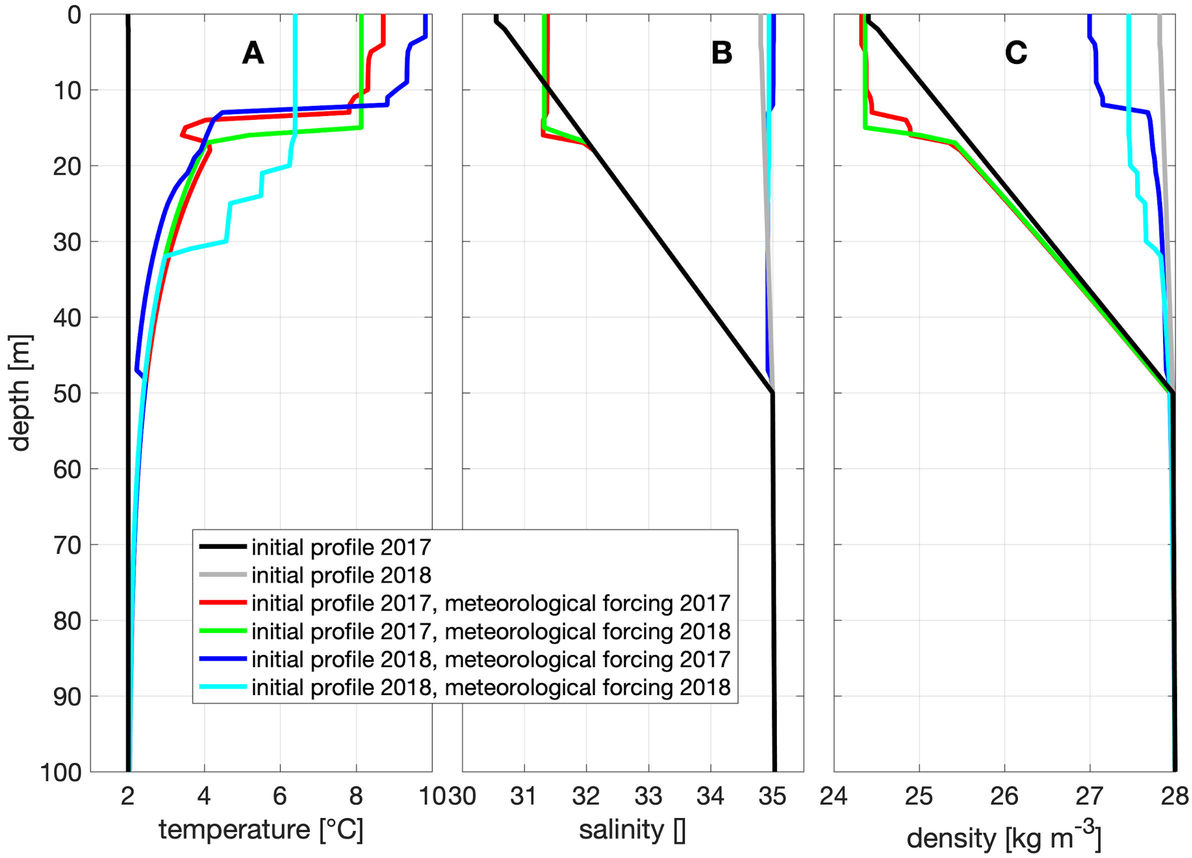

Figure S6: **Results from 1-dimensional mixed layer model.**

A temperature [°C], **B** salinity, and **C** density [kg m^{-3}] profiles at the beginning (15-May-2017/2018 shown in black/gray) and end (01-Aug-2017/2018 shown in colors) of the four PWP model runs (see methods section).

Note that the final profiles resulting from the different initial profiles are very different. By contrast, the surface forcing only introduces minor changes to the final profiles. In all cases, the surface forcing does not significantly affect the ocean below the top 30 m. Since the surface heat fluxes are positive during this time period, the surface ocean warms, which decreases the near-surface density.
