# Supplementary material for: Sea-ice derived meltwater stratification slows the biological carbon pump: results from continuous observations
Source: Nat Commun. 2021 Dec 15;12:7309. doi: 10.1038/s41467-021-26943-z (PMC8674288; doi:10.1038/s41467-021-26943-z)
Supplement: Supplementary file 3 — Description of Additional Supplementary Files [file 41467_2021_26943_MOESM3_ESM.pdf]

File Name: Supplementary_Data_1.xlsx
Description: Excel table with data according to Table S1 that is not available online. The file also contains Tables S1, S2, and S3 as separate sheets.
